# Health-Related Quality of Life of the General German Population in 2015: Results from the EQ-5D-5L

**DOI:** 10.3390/ijerph14040426

**Published:** 2017-04-16

**Authors:** Manuel B. Huber, Julia Felix, Martin Vogelmann, Reiner Leidl

**Affiliations:** 1 German Research Center for Environmental Health, Institute for Health Economics and Health Care Management, Helmholtz Zentrum München, Postfach 1129, Neuherberg 85758, Germany; julia.felix@helmholtz-muenchen.de (J.F.); leidl@bwl.lmu.de (R.L.); 2 Wort & Bild Verlag Konradshöhe GmbH & Co. KG, Baierbrunn 82065, Germany; martin.vogelmann@wortundbildverlag.de; 3 Munich Center of Health Sciences, Ludwig-Maximilians-University, Ludwigstr. 28 RG, Munich 80539, Germany

**Keywords:** health-related quality of life, EQ-5D-5L, population survey, Germany, 2015

## Abstract

The EQ-5D-5L is a widely used generic instrument to measure health-related quality of life. This study evaluates health perception in a representative sample of the general German population from 2015. To compare results over time, a component analysis technique was used that separates changes in the description and valuation of health states. The whole sample and also subgroups, stratified by sociodemographic parameters as well as disease affliction, were analyzed. In total, 2040 questionnaires (48.4% male, mean age 47.3 year) were included. The dimension with the lowest number of reported problems was self-care (93.0% without problems), and the dimension with the highest proportion of impairment was pain/discomfort (71.2% without problems). Some 64.3% of the study population were identified as problem-free. The visual analog scale (VAS) mean for all participants was 85.1. Low education was connected with significantly lower VAS scores, but the effect was small. Depression, heart disease, and diabetes had a strong significant negative effect on reported VAS means. Results were slightly better than those in a similar 2012 survey; the most important driver was the increase in the share of the study population that reported to be problem-free. In international comparisons, health perception of the general German population is relatively high and, compared with previous German studies, fairly stable over recent years. Elderly and sick people continue to report significant reductions in perceived health states.

## 1. Introduction

One key goal of health care provision is to improve health-related quality of life (HRQoL) [1]. HRQoL describes a multidimensional construct that includes physical, mental, functional, and social factors determining quality of life [2]. In recent years, HRQoL has gained considerable importance in the assessment of health care interventions. Furthermore, HRQoL is considered to be a fundamental measure of population health [2,3,4]. In an aging society, with increasing prevalence of chronic diseases, collecting mortality and morbidity data is no longer sufficient to assess the health impact of disease [5]. Therefore, instruments are used to measure HRQoL at the population level. One widely used and validated instrument is the EuroQol five-dimension (EQ-5D) questionnaire [6,7,8,9,10]. The EQ-5D-5L is currently available in 171 languages and has been used to measure HRQoL in population surveys from various countries including the USA, Canada, the UK, and Germany [11,12,13,14,15]. In a nationwide survey from Germany in 2015, the EQ-5D-5L questionnaire was also used to measure the HRQoL of a representative sample of the general German population. The aim of this study is to complement previous population studies by analyzing the latest results on general health perception in Germany. Focusing on the impact of age, sex, socioeconomic variables, and chronic disease, this evaluation allows the identification of possible health trends and enables comparisons of health perception across countries. Results of this study can serve as evidence for policy makers and as an up-to-date general population reference for comparison of clinical populations.

## 2. Materials and Methods

Our dataset is based on an annual survey of the general German population conducted by the IFAK research institute [16,17,18,19] on behalf of Wort & Bild Verlag (W&B). The survey’s goal is to gather information regarding trends in health care, but the survey also includes questions on the health status of the German population. Selected participants have to be part of the German population and at least 14 years of age. Personal interviews for this survey were conducted from September to October 2015. The EQ-5D-5L questionnaire was completed in written form by participants themselves. To retrieve a representative sample of the population, the W&B survey used a random-route procedure based on 516 sample points, structured by a specific BIK code (named after BIK Aschpurwis + Behrens GmbH, Hamburg, Germany), a demographic and geographic classification system that has also been used in past surveys. More details on the sampling system can be found elsewhere [16].

### 2.1. EQ-5D-5L

The EQ-5D-5L is a generic instrument measuring HRQoL and consists of two parts: first, a descriptive system and, second, a visual analog scale (VAS). The descriptive system includes five dimensions (mobility, self-care, usual activity, pain/discomfort, anxiety/depression) to describe the health status of survey subjects. The VAS is used to assess general health and is a continuous response scale ranging from 0 to 100, where 0 describes the worst and 100 the best possible condition [6,20]. Every dimension of the EQ-5D-5L includes five answer levels, covering no problems (1) to extreme problems (5). Based on this, the descriptive score “11111” represents the best, problem-free health state and “55555” the worst. The EQ-5D-5L is an updated version of the EQ-5D-3L questionnaire which includes only three answer levels for each dimension. Advantages of the EQ-5D-5L, compared with the EQ-5D-3L, include a greater variety of reported health states whereby, inter alia, ceiling effects are reduced when used in a general population survey [11]. Psychometric properties of the EQ-5D-3L and -5L have been evaluated across different diseases [8,21,22,23], and the questionnaire is used by a wide variety of well-respected institutions including the NHS [24].

### 2.2. Data Analysis

All data analysis was conducted independently with SAS 9.3 (SAS Institute Inc., Cary, NC, USA) and R 3.3.0 (R Foundation for Statistical Computing, Vienna, Austria) by two different researchers. Because of the annual assessment of health perception by the W&B survey and the lack of any major health-related event in Germany, we expected that health perception would remain quite stable compared with the previous three years. Demographic and socioeconomic variables such as education, income, and occupation [25] have been found to influence quality of life measured by EQ-5D-3L in the general population [26,27,28]. Accounting for these important determinants in the analysis of this EQ-5D-5L survey, our study will evaluate the influence of the variables age (classified by age groups), sex, educational background (“low”: in education, high school with/without apprenticeship; “medium”: middle school; “high”: grammar school with/without university attendance), occupation, and information about chronic diseases. To evaluate chronic diseases, participants were asked to select up to six different diseases they currently are affected by, from a list. Considering distributional assumptions, a linear model and the maximum likelihood estimation were used to examine the impact of different parameters on VAS means. To account for patient heterogeneity, we refrained from using value sets [29] and focused on VAS scores reported by respondents. To evaluate the stability of observed health states, we employed a component analysis developed by Kitagawa in 1955 [30]. The component analysis includes both the change in health state composition (=the share of people in each health state) as well as changes in valuation for each health state (e.g., how VAS means change for each group and year). We compared data from this survey with survey data from 2012, the first year in which the EQ-5D-5L was used in W&B surveys. Comparison was restricted to health states reported by at least five respondents in each sample. The difference in VAS means from 2015 and 2012 is split into a case-mix component as well as a valuation component and interaction term (Equation (1)).

Equation (1). Component analysis for EQ-5D-5L health valuation based on Kitagawa (1955) [30]

$$\begin{array}{c} VAS_{A}-VAS_{B}=\sum_{i}VAS_{Bi}\;\left(p_{Ai}-p_{Bi}\right)\\ +\sum_{i}p_{Bi}\;\left(VASp_{Ai}-VASp_{Bi}\right)+\left(p_{Ai}-p_{Bi}\right)(VASp_{Ai}-VASp_{Bi}) \end{array}$$

*p* = % in each health state
*i* = health state
*A*, *B* = group year *A*, year *B*
∑ = sum over all groups *i*

## 3. Results

### 3.1. Characteristics of the Study Population

The total survey response rate was high and reached 71.8%. The most frequent reason for non-response was absence of household members in 261 cases. A total of 2055 participants attended the survey, but only 2040 questionnaires could be evaluated because 15 participants had missing health perception data. Overall, more women participated in the survey. The average age was approximately 47 years. More than 60% of participants had medium or high education. The majority were employed full-time or part-time (Table 1).

### 3.2. Descriptive System

Among the five dimensions, the fewest problems were reported for self-care (7.0%) and the most for pain/discomfort (28.8%), followed by mobility (18.3%). Severe problems in any dimension were very rare (1.4%). A majority of 64.3% were reported to be problem-free (11111).

### 3.3. VAS

In accordance with the descriptive system, VAS means were mostly within the upper range of the scale. The VAS mean for all participants was 85.1.

#### 3.3.1. Age and Sex

**Figure 1:**
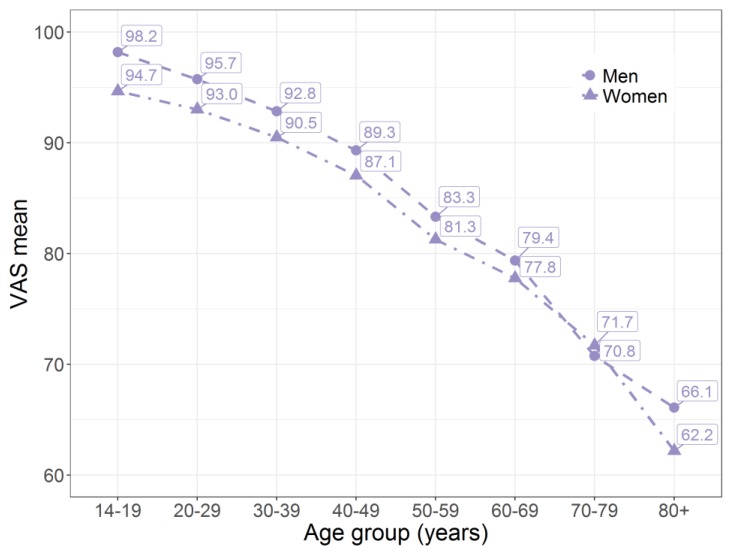
VAS means stratified by age group and sex.

**Figure 2:**
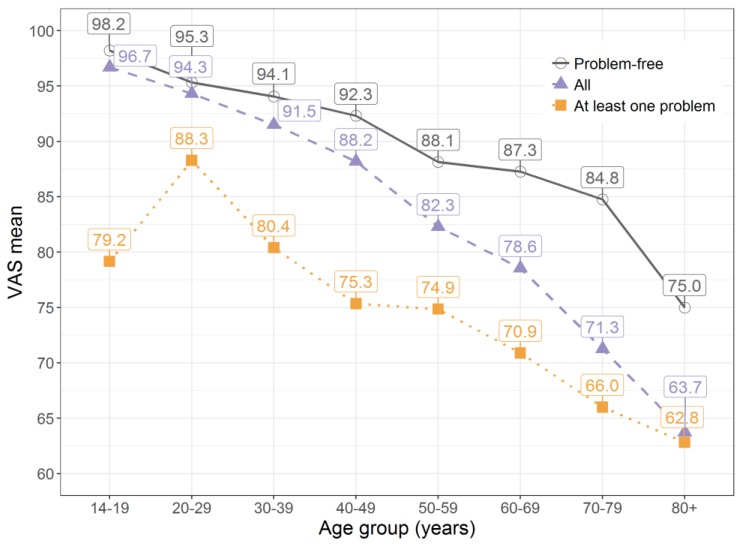
VAS means stratified by age group and health status. Note: Problem-free: descriptive score “11111”; All: VAS means of the entire study population; At least one problem: sample with at least one problem in at least one dimension.

Men had a slightly higher VAS mean than women (86.4 vs. 83.9), whereas on average, women were more than 1 year older than men. The marginally higher VAS mean for men was also observed across age groups (Figure 1). Only in age group 70–79 years did women have a higher VAS mean than men (71.7 vs. 70.8). Overall, the VAS means decreased with age (Figure 1 and Figure 2).

**Figure 3:**
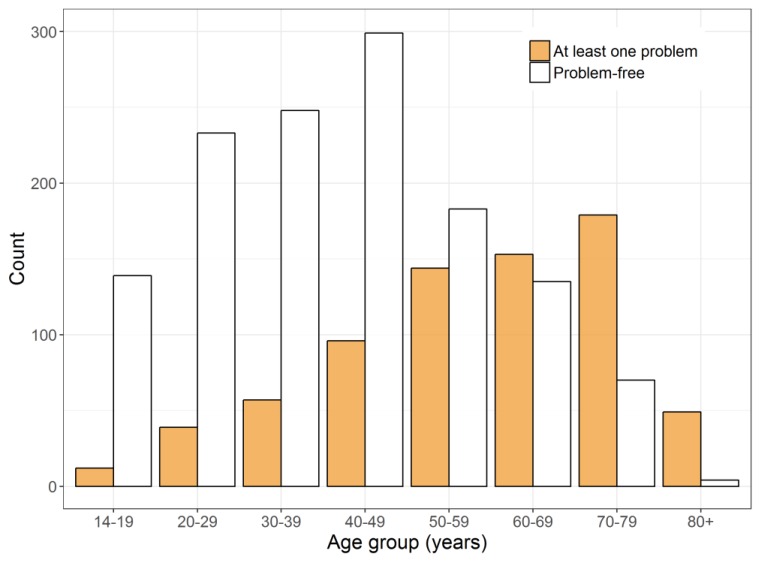
Sample share stratified by age group and health status.

The VAS means also varied depending on whether there were problems reported in the individual dimensions or not. The VAS mean for participants in the problem-free health state (11111) was 92.3. For participants with at least one reported problem, the VAS mean was 20 points less. The VAS distribution by health status and age is illustrated in Figure 2. From age group 60–69 years onwards, the share of participants with at least one problem was greater than the share of people who reported to be problem-free (Figure 3).

Overall, the VAS mean of the 70- to 79-year-old participants was 25 points below the VAS mean of the 14- to 19-year-old participants. This tendency was also observed for participants with at least one problem. Only the mean VAS score for those younger than 19 years (n = 12) was markedly lower. From age group 70–79 years to 80+ years, a big decrease in VAS means of around 10 points was reported. Furthermore, the percentage of the study population without any health restrictions became smaller with increasing age. Although 92.1% of age group 14–19 years were problem-free, only 7.6% of age group 80+ years reported no problems.

#### 3.3.2. Education

**Figure 4:**
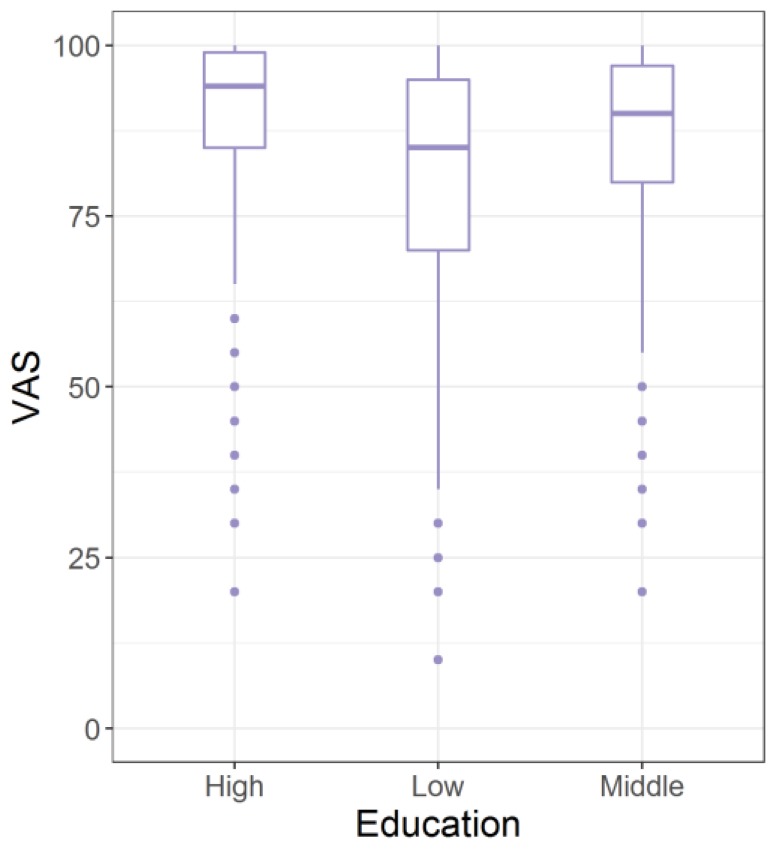
Boxplots for the distribution of VAS means by educational background.

VAS means increased with higher educational background but variance was high (Figure 4).

#### 3.3.3. Chronic Diseases

The most often stated chronic diseases were musculoskeletal disorders (n = 143), hypertension (n = 141), and diabetes (n = 65). Table 2 shows the results of the multivariate linear model evaluating the impact of sex, age, educational background, and disease affliction on VAS score. The density plot of the models’ residuals was hardly skewed (not reported here), so no transformation was pursued considering parameter interpretation. Although female sex and low educational background were associated with slight deductions, depression and heart disease had a strong and significant negative impact. Diabetes and arthrosis were also related to substantial deductions, whereas thyroid disease and medium educational background had no significant impact.

### 3.4. Component Analysis

**Figure 5:**
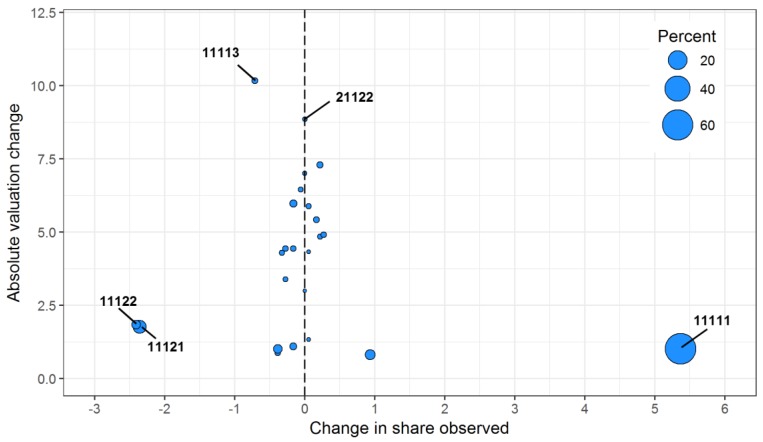
Component analysis based on Kitagawa (1955) [30]. Note: Most significant health states are labelled. Percent refers to the mean share of participants in the respective health state across both years.

A total of 24 health states were observed by five or more respondents in both samples, covering 89.2% of the 2012 as well as the 2015 respondents. With a VAS mean of 88.1 in 2015 and 86.3 in 2012, a slight increase in VAS mean was observed by 1.8 on the 100 VAS scale. This is far below any minimal clinical relevance. Decomposition of overall change shows that the case-mix component, i.e., contributions from different health states reported, was 0.7 on the 100 VAS scale. The valuation component reached 1.1 on the 100 VAS scale, whereas the interaction term was at 0.1. Details of valuation changes vs. changes in shares observed show a 5.4% increase in the problem-free health state (11111) but small valuation change (Figure 5), but only referring to about two thirds of the respondents in the 2012 subsample analyzed here. Other health states make only minor contributions. Taken together, a marginal but clinically non-relevant increase was observed; thus, health state valuation remained quite stable.

## 4. Discussion

Evaluating the health status of a representative sample of the general German population from 2015 leads to several important results. The majority of participants reported no limitations in the five dimensions of the EQ-5D-5L. Women reported slightly lower HRQoL than men. A stronger negative association was seen for low education compared with high education. Furthermore, as expected, HRQoL deteriorates with increasing age. Extreme limitations were only rarely reported. Specific chronic diseases have a strong and significant negative impact on general health status.

### 4.1. Age

VAS means decrease significantly with age. This might indicate an increase in disease-related restrictions as people get older. Hinz et al. [13] observed an almost linear decrease in HRQoL, measured by sum score, with increasing age. This is in line with previous studies in which HRQoL was measured by EQ-5D [13,31,32] or other measuring instruments [13,33,34,35]. The big decrease in VAS mean from age group 70–79 years to 80+ years for participants without any restrictions might result from health-related problems that are not covered by the five dimensions. However, the small sample size (age 80 + years, n = 4) may also be a reason for distortions in this age group.

### 4.2. Sex

Men had higher VAS means than women in every age group except 70–79 years. The international overview by Szende et al. [32] displays similar results using the EQ-5D-3L, and a number of other studies indicate this gender-specific trend irrespective of the measurement method [11,27,36,37,38]. Interestingly, we observed the same trend reversal for age group 70–79 years as did McCaffrey et al., 2016 [39]. In both studies, these were the only age groups in which women reported better health perception than men. In general, the differences between the genders are comparatively moderate and far below the limits that are considered as minimal clinically important differences [40].

### 4.3. Education

The linear model shows a significant negative impact of low education on VAS means. This is also observed in previous studies [17,41,42,43]. Education cannot explain all valuation change but, if the educational distribution of a similar study from 2006 [18], in which the proportion of participants with low education was 7.3 percentage points higher than in this study, were to be applied to the explanatory VAS model in this study, the VAS mean for the entire study population would be slightly lower at 84.7 (vs. 79.2 in 2006).

### 4.4. Chronic Diseases and VAS

Another negative association was found for chronic diseases and VAS means (Table 2). In particular, depression, heart disease, and diabetes seem to have strong negative effects on HRQoL. Golicki et al. [44] reported between 15.7 and 23.2 points lower VAS means for participants with type 2 diabetes compared with the general population. A study from Korea [45] also shows a decrease of 9.6 points for VAS means in people with coronary heart disease. Recent research from China [46] regarding the HRQoL of mentally ill participants compared with older participants without psychological disorders shows estimators of −10.7 for heart disease and −8.3 for mental illness. These results underscore the outcomes of our study.

### 4.5. Comparison with Other Studies

The mean VAS score in this study is 85.1, the average age is 47.3 years, and the percentage of female participants is 51.6%. A range of comparative studies exist using the EQ-5D-3L. However, the descriptive system of the EQ-5D-3L is less differentiated than the 5L answer version. An earlier study by W&B Verlag reported a VAS mean of 79.2 for a study population with 54.4% women and an average age of 46.4 years in 2006 [18,19]. In addition to the increase in HRQoL since 2006 shown in comparison with VAS values in this study, surveys by the Bertelsmann Gesundheitsmonitor [47] indicate a considerable improvement in satisfaction with health care provision. In 2006, only 36% of people with statutory health insurance were rather or very satisfied, compared with 61% in 2015.

The review by Szende et al. [32] enables international comparison. VAS means of 75.3 are reported for Argentina and 83.7 for Denmark; the value for Germany is 77.3. It should be noted that the German survey data [38] in this review date back to 2001/2003, and the average age (48.1 years) as well as the percentage of women (51.8%) were slightly higher. A Swedish survey with 49,169 participants living in mainly urban areas in Sweden reports a VAS mean of 79.5 [38]. The study dates back to 2004/2006, the average age is 46.2 years, and 56.3% of participants were female. A study from France [48] reported a VAS mean of 77.0 (average age 46.1 years; share of women 51.7%) but included 51.8% of participants with low education (the term was not specified further).

Only a few comparative studies exist for the EQ-5D-5L as a measure of population health. One of the first German EQ-5D-5L studies in 2011 [13] does not report any VAS values, as does a study from Poland [49]. This aggravates overall HRQoL assessment, as the five dimensions of the descriptive system do not capture all aspects of disease equally well [50,51,52].

The VAS values in this study can also be compared with earlier and methodologically identical studies from 2012, 2013, and 2014, which show relatively high VAS means (83.3; 84.9; 84.8) [16]. The comparison with 2012 data (Figure 5) revealed that health state valuation remained quite stable. Only the share of people reporting no problems (= 11111) increased by 5.4%. Further research has to determine to what extent this difference and possible trends are based on sample heterogeneity.

There are four comparable international studies for the EQ-5D-5L. The study by Garcia-Gordillo et al. [53] states a VAS mean of 75.7 (average age not available; share of women 54.1%) in the general Spanish population. Feng et al. [11] report a VAS mean of 78.4 for England. The average age was 51.6 years (author correspondence), which is 4 years higher than in this study. Moreover, the percentage of women participating in the study was 59.3%. Another study with 4406 participants from Alberta, Canada, reports a VAS mean of 79.0, despite a higher proportion of men (50.1%) and lower average age (46.1 years) [54]. It is surprising that the VAS difference between the youngest age group (18–29 years: 83) and the oldest age group (80+ years: 75) is merely 8 points. This is considerably lower than the 30-point difference in our study. However, one distinctive difference between the Canadian and this study is the selection procedure. The response rate of the Canadian telephone interviews was only 31.7%. Strong self-selection cannot therefore be excluded. It is possible that sicker people with lower HRQoL are more likely to participate in such surveys. McCaffrey et al. (2016) reported a mean VAS score of 78.6 for a large community sample from South Australia [39]. The mean age in their study was 46.3 years and 51.1% of participants were female. Despite these methodological differences, the national as well as the international studies indicate a relatively high overall perception of HRQoL in Germany that has been relatively consistent over recent years.

### 4.6. Limitations and Strength

One limitation of this study is the small sample size of participants with extreme problems and the large number of people who were reported to be problem-free (61.5% vs. below 50% in most other studies). It seems obvious that seriously ill people with low HRQoL are seldom found at home because they are in hospital or unable to participate for other reasons. For the evaluation of EQ-5D-5L health states with extreme problems, further studies based on adapted selection processes would be useful. A weighting of data in accordance with census data has not taken place, but the deviation from W&B data was small (<1% in the majority of age groups). One strength of this study lies in the annual repetition of the W&B survey, the database for this study. The relatively similar database enables good comparability of data across single years as long as the same version of the EQ-5D is used. Furthermore, this study is one of the first studies to evaluate the EQ-5D-5L and especially the VAS for measuring the health status of the general German population. The utilized random-route procedure to select the study population ensures high representability of the database.

## 5. Conclusions

Compared with previous studies, the health perception of the general German population remains relatively stable. A slight increase in VAS values was observed. Older people with specific diseases perceive significant decreases in HRQoL, especially those affected by depression, heart disease, or diabetes. Health perception of the general German population in 2015 is relatively high compared with (mostly older) population surveys from other countries. Changes in population health can be detailed using component analysis techniques.

## Figures and Tables

**Table 1:** Sample characteristics.

Characteristics		n	%
**Sex**	male	987	48.38
	female	1053	51.62
**Mean age (years)**	total: 47.32 (±18.17)		
	males: 46.77 (±18.08)		
	females: 47.84 (±18.24)		
**Age groups (years)**	<19	151	7.40
	20–29	272	13.33
	30–39	305	14.95
	40–49	395	19.36
	50–59	327	16.03
	60–69	288	14.12
	70–79	249	12.21
	80+	53	2.60
Education *	low	822	40.29
	medium	785	38.48
	high	433	21.23
**Occupation**	full-time	1012	49.61
	part-time	253	12.40
	leave (parental leave, military/civil service)	12	0.59
	unemployed	53	2.60
	pension	452	22.16
	housewife	70	3.43
	undergoing training	96	4.71
	no information	92	4.51

* Low education: in education, high school with/without apprenticeship; medium education: middle school; high education: grammar school with/without university attendance.

**Table 2:** Linear model results, VAS as dependent variable.

Coefficient	Estimator	SE	Pr (>|t|)
(Intercept)	107.15	1.09	***
Sex (female)	–1.56	0.54	**
Age	–0.33	0.02	***
Low education	–2.27	0.73	**
Medium education	0.50	0.71	
High education	Reference		
Depression	–17.57	2.55	***
Hypertension	–7.48	1.13	***
Migraine	–5.62	2.21	*
Diabetes	–12.45	1.56	***
Musculoskeletal disorders	–8.95	1.09	***
Thyroid disease	–2.78	2.14	
Heart disease	–14.51	1.92	***
Gastrointestinal diseases	–4.20	2.02	*
Rheumatism	–8.76	2.39	***
Arthrosis	–9.90	1.95	***

SE: Standard error; Level of significance: * *p* < 0.05, ** *p* < 0.01, *** *p* < 0.001.
